# Magnetic resonance T1w/T2w ratio and voxel-based morphometry in multiple system atrophy

**DOI:** 10.1038/s41598-021-01222-5

**Published:** 2021-11-04

**Authors:** S. Ponticorvo, R. Manara, M. C. Russillo, R. Erro, M. Picillo, G. Di Salle, F. Di Salle, P. Barone, F. Esposito, M. T. Pellecchia

**Affiliations:** 1 Neuroscience Section, Department of Medicine, Surgery and Dentistry, Scuola Medica Salernitana, Center for Neurodegenerative Diseases (CEMAND), University of Salerno, 84131 Salerno, Italy; 2 Neuroradiology Unit, Department of Neurosciences, University of Padua, Padua, Italy; 3 Classe di Scienze Sperimentali, Scuola Superiore di Studi Universitari e Perfezionamento Sant’Anna, Pisa, Italy; 4 Department of Advanced Medical and Surgical Sciences, University of Campania “Luigi Vanvitelli”, Naples, Italy

**Keywords:** Biomarkers, Neurology

## Abstract

Diagnosis of multiple system atrophy (MSA) may be improved by using multimodal imaging approaches. We investigated the use of T1-weighted/T2-weighted (T1w/T2w) images ratio combined with voxel-based morphometry to evaluate brain tissue integrity in MSA compared to Parkinson’s disease (PD) and healthy controls (HC). Twenty-six patients with MSA, 43 patients with PD and 56 HC were enrolled. Whole brain voxel-based and local regional analyses were performed to evaluate gray and white matter (GM and WM) tissue integrity and mean regional values were used for patients classification using logistic regression. Increased mean regional values of T1w/T2w in bilateral putamen were detected in MSA-P compared to PD and HC. The combined use of regional GM and T1w/T2w values in the right and left putamen showed the highest accuracy in discriminating MSA-P from PD and good accuracy in discriminating MSA from PD and HC. A good accuracy was also found in discriminating MSA from PD and HC by either combining regional GM and T1w/T2w values in the cerebellum or regional WM and T1w/T2w in the cerebellum and brainstem. The T1w/T2w image ratio alone or combined with validated MRI parameters can be further considered as a potential candidate biomarker for differential diagnosis of MSA.

## Introduction

Multiple system atrophy (MSA) is an adult-onset progressive neurodegenerative disorder featured by autonomic failure, parkinsonism and cerebellar ataxia with a prevalence of about 5 cases per 100.000. Oligodendroglial cytoplasmatic inclusions consisting of misfolded a-synuclein are required for a definite diagnosis of MSA on postmortem examination^1^. Neuronal loss, pathologic inclusions, iron accumulation and reactive astrogliosis are observed in the striatonigral and olivopontocerebellar systems^2,3^. MSA can be clinically classified into the parkinsonian variant (MSA-P) and the cerebellar variant (MSA-C) based on the predominant motor features which is dependent on the distribution of pathology within the basal ganglia and cerebellum^1^. However, the predominant motor features can change with time and the variability of the severity and regional distribution of pathological process accounts for a spectrum of disease.

The differential diagnosis between MSA, particularly the parkinsonian subtype, and Parkinson’s disease (PD), the most frequent α-synucleinopathy, may be difficult due to the presence of common clinical features, as demonstrated by the relatively high rate of misdiagnosis at post-mortem evaluation^4^.

Previous studies suggested a role of different imaging techniques in aiding the differential diagnosis between PD and MSA. Atrophy on Magnetic Resonance Imaging (MRI) of putamen, middle cerebellar peduncle, pons, or cerebellum on conventional MRI and hypometabolism on FDG- PET in the putamen, middle cerebellar peduncle, pons, and cerebellum are already included as additional features for a diagnosis of possible MSA in the current diagnostic criteria^1^. Moreover, it has been shown that suboptimal accuracy of neuroradiological diagnosis may be improved by the use of multimodal imaging approaches and advanced MRI techniques^5^.

So far, the ratio of the signal intensity of the T1-weighted and T2-weighted (T1w/T2w) MRI images, has been used as semi-quantitative measure for myelin content in gray matter^6^. Compared to other quantitative MRI techniques, it has the advantage that images can be easily acquired during routine clinical examination and without complex modeling of the MR signal, with high spatial resolution and sensitivity to neurodegenerative changes^7–9^.

In disorders with a strong demyelinating component (as e.g. in multiple sclerosis), the T1w/T2w ratio has been found to be lowered in pathologically vulnerable regions^10,11^. However, recent studies have also pointed out on a different interpretation of the T1w/T2w ratio, suggesting that this measure may also reflect axon and dendrite density or iron content^7^. Consistently, increased T1w/T2w ratio has been recently found in the substantia nigra pars compacta of PD patients compared with healthy controls^12^.

Since MSA-related pathology involves oligodendroglial and neuronal loss, with astroglial and microglial activation and increased iron content, in this study, we have performed a multimodal evaluation of tissue integrity by assessing both T1w/T2w ratio images and voxel-based morphometry (VBM) on gray and white matter (GM and WM) brain compartments. A multi-parameter analysis was performed with the aims of unveiling tissue damage in MSA patients as compared to healthy controls (HC) and PD patients and for assessing a new potential candidate MRI biomarker for differential diagnosis with PD.

## Methods

### Subjects

Twenty-six patients with probable MSA according to current diagnostic criteria [14 with the parkinsonian variant (MSA-P) and 12 with the cerebellar variant (MSA-C)], 43 patients with idiopathic PD and 56 healthy controls (HC) participated to the current study. Motor disability was assessed with the Unified MSA Rating Scale, part two (UMSARS-II) and the Unified PD Rating Scale, part three (UPDRS-III) in MSA and PD patients, respectively. In order to perform an additional exploratory analysis of clinico-radiological correlates in MSA, we also looked at specific items from the UMSARS I, and specifically we used item 1 to 6 total score for difficulties with daily activities, item 7 and 8 scores for walking difficulties and falls respectively, and item 8 to 12 total score for dysautonomia. Demographic and clinical data of enrolled subjects are reported in Table 1. The study was approved by the local Ethics committee—Comitato Etico Campania Sud—and all participants signed informed consent. The study was conducted in accordance with the Declaration of Helsinki principles.

**Table 1:** Demographic and clinical findings of enrolled subjects.

	MSA (n = 26)	PD (n = 43)	HC (n = 56)	*p*
Age, ys (mean ± SD)	59.5 ± 6.7	64.3 ± 7.9	62.9 ± 9.1	ns
Gender (M/F)	15/11	26/17	34/22	ns
Disease duration, ys (mean ± SD)	3.6 ± 1.3	4.4 ± 2.4	–	ns
H&Y stage (mean + SD)	2.5 ± 0.5	1.8 ± 0.54	–	ns
UPDRS-III (mean ± SD)	–	17.6 ± 9.6	–	
UMSARS-I (mean ± SD)	22.07 ± 6	–	–	
UMSARS-II (mean ± SD)	24.07 ± 6.9	–	–	
UMSARS-IV (mean ± SD)	2.7 ± 0.8	–	–	

### MRI acquisition

All brain imaging data were acquired on a 3 T MRI scanner (MAGNETOM Skyra, Siemens, Erlangen Germany) operated with a 20-channel head and neck coil. The imaging protocol consisted of a 3D anatomical T1-weighted (T1w) Magnetization Prepared RApid Gradient Echo (MPRAGE) sequence with repetition time (TR) = 2400 ms and echo time (TE) = 2.25 ms, spatial resolution = 1 × 1 × 1 mm3, matrix size = 256 × 256, anterior–posterior phase encoding direction, generalized autocalibrating partially parallel acquisitions (GRAPPA) factor of 2 in phase-encoding direction and a 3D T2-weighted (T2w) Sampling Perfection with Application optimized Contrast using different angle Evolutions (SPACE) sequence with TR = 3200, TE = 408 ms, variable flip angle, resolution = 1 × 1 × 1 mm3, matrix size = 256 × 256, anterior–posterior phase encoding direction, GRAPPA factor of 2 in phase-encoding^9^.

### MRI data processing

For VBM analysis tissue probabilistic maps were obtained from T1w images and used to evaluate differences in terms of grey and white matter (GM and WM) atrophy. T1w native space images of each subject were segmented into GM and WM and normalized to MNI standard space using DARTEL algorithm^13^. Then the resulting tissue (GM/WM) probabilistic maps were modulated by the Jacobian determinants of the deformations to account for local compression and expansion due to linear and non-linear transformation^14^ and then smoothed with a Gaussian kernel of 6 mm FWHM. For the group analysis a group mask was created for each tissue, averaging and then binarizing with a threshold of 0.2 all the smoothed GM/WM maps of the HC subjects. Total intracranial volume (ICV) was also calculated for each subject as the sum of the three main brain tissue volumes (GM, WM and CSF).

In order to obtain semi-quantitative maps markers of myelin content, before the validated preprocessing^6^, T1w and T2w images were corrected for intensity nonuniformity with the bias correction tool implemented in the unified segmentation^14^ and available in SPM12. Then the T2w images were linearly registered to the T1w images using the FSL tool FLIRT^15,16^ for estimating and applying a rigid-body affine transformation with 6 degrees of freedom and cubic spline interpolation to minimize the WM and CSF contamination of GM voxels^6^. T1w/T2w maps were obtained using FSLMATHS to divide the T1w volumes by the corresponding aligned T2w ones. For the group analysis the DARTEL algorithm with the same group template and deformation fields as calculated for VBM analysis were used to normalize the T1w/T2w ratio maps to the standard MNI space. During the normalization procedure, T1w/T2w maps were smoothed with a Gaussian kernel of 6 mm FWHM.

### Statistical analysis

The whole brain maps (GM and WM probability tissue maps and T1w/T2w ratio maps) were compared between groups in a voxel-based full factorial analysis as implemented in SPM12. Particularly a general linear model was used considering one factor of three levels for the group (MSA, PD, HC) and two (age and sex) and three (age, sex and ICV) covariate factors respectively for T1w/T2w and GM or WM analyses. Voxels were considered significant with *p* < 0.05 after family-wise error correction (FWE) for multiple comparisons as implemented in SPM12.

A post-hoc regional analysis was also performed by extracting the mean parameter values (GM and WM probability and T1w/T2w ratio) in the voxels of detected differences in the voxel-based analysis and in the substantia nigra pars compacta (SN) individuated using masks freely available at https://github.com/apoorvasafai/NMS-SNc-atlas. The regional values were compared between groups (MSA, PD, HC) and MSA subgroups (MSA-C, MSA-P) with a two-sample t-test after correcting for age with linear regression.

Receiver operating characteristic (ROC) curve analysis was then performed to evaluate the ability of the MRI parameters to discriminate between MSA and HC, MSA and PD and MSA-P and PD. For this analysis, a generalized linear model with binomial distribution and logit link function as setting parameters was first computed on the regional values of GM or WM and T1w/T2w. For each ROI, the ROC curve was calculated on the obtained predictive values, allowing to calculate the corresponding area under the curve (AUC), confidence interval, and *p*-values. The optimal cutoff point (and corresponding sensitivity and specificity) was determined using the Youden method.

Correlations between MRI parameters and clinical variables were checked with the Spearman’s rank correlation coefficient both considering the whole MSA group and the subgroups (MSA-P and MSA-C); a 5% level of significance was used for all tests.

## Results

The three groups did not differ in age and sex distribution (Table 1). Patients with MSA and PD had similar disease duration (Table 1). Disease severity, as assessed by UPDRS-III and UMSARS, is reported in Table 1.

**Figure 1:**
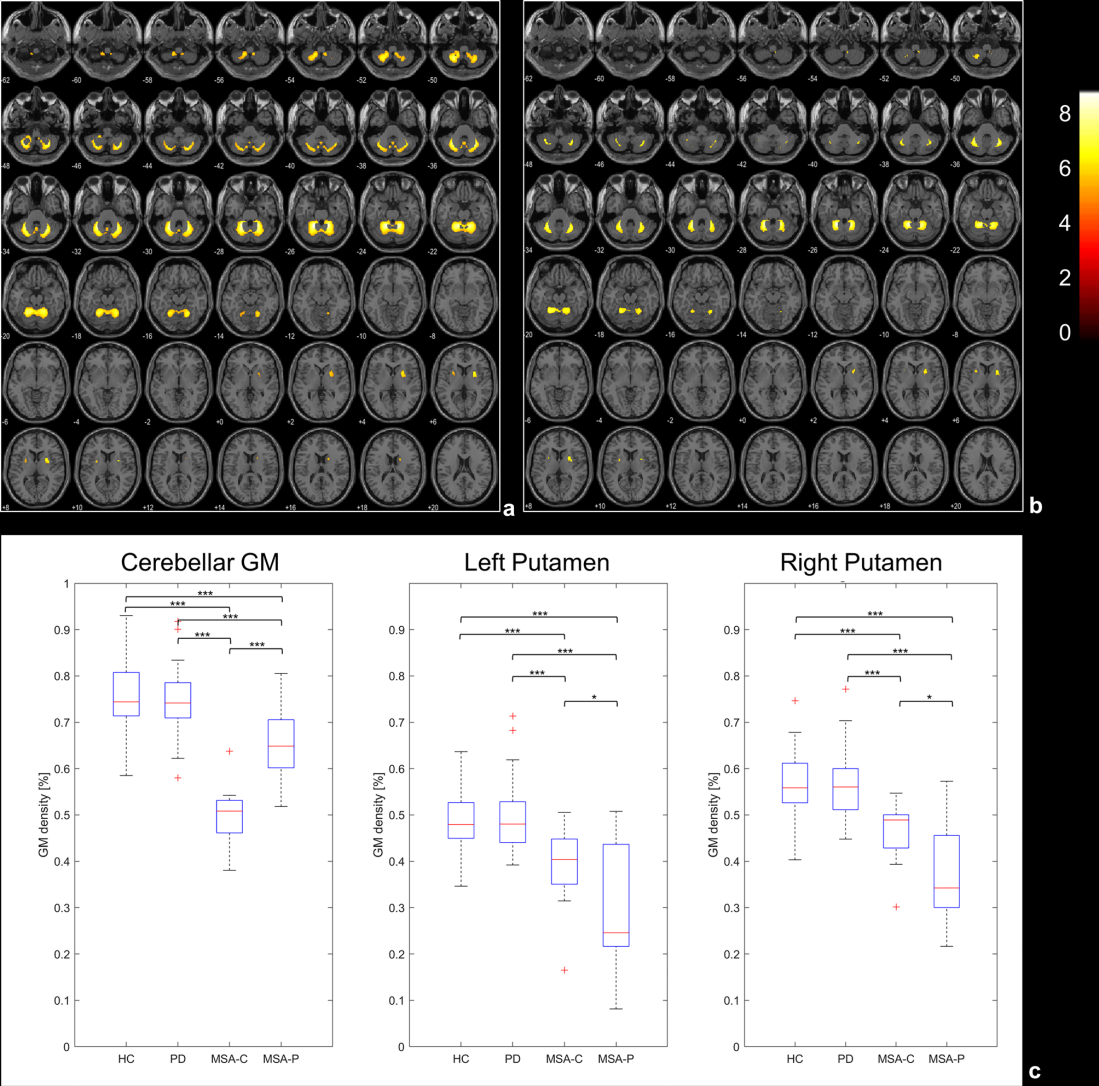
Upper panel t-maps of the voxel-wise GM comparisons respectively between HC and MSA (**a**) and PD and MSA (**b**). Statistical threshold is set to *p* < 0.05 after FWE correction for multiple comparison. Lower panel box-plots of the GM density distribution in the significant clusters (from the comparison HC vs MSA) for each group, and results of the post-hoc t-tests on the regional values (**c**). *p < 0.05, **p < 0.01, ***p < 0.001.

Compared to HC, MSA patients showed reduced GM volume in bilateral putamen (left cluster size = 128 voxels, right cluster size = 1143 voxels) and in an extended cluster in the cerebellar gray matter (cluster size = 379,270 voxels) (Fig. 1a). When comparing MSA versus PD patients, the former showed reduced GM volume in two clusters of bilateral putamen (left cluster size = 119 voxels, right cluster size = 749 voxels) and in an extended area of the cerebellar gray matter (cluster size = 134,350 voxels) (Fig. 1b). No significant differences were detected when comparing PD versus HC. Post-hoc regional analyses displayed significant between-group differences, between MSA-P and both PD and HC, and between MSA-C and both PD and HC (Fig. 1c).

**Figure 2:**
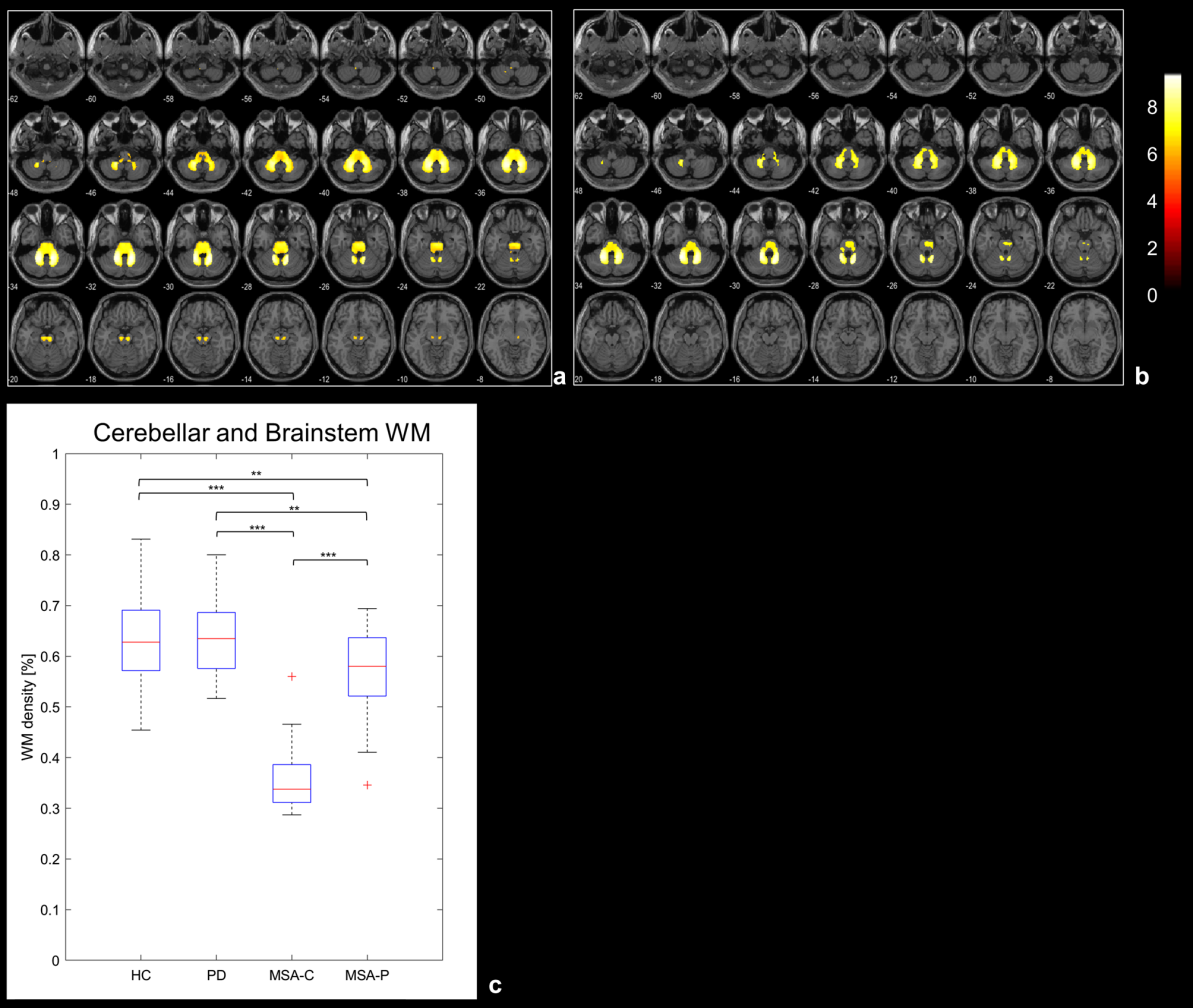
Upper panel t-maps of the voxel-wise WM comparisons respectively between HC and MSA (**a**) and PD and MSA (**b**). Statistical threshold is set to *p* < 0.05 after FWE correction for multiple comparison. Lower panel box-plots of the WM density distribution in the significant cluster (from the comparison HC vs MSA) for each group, and results of the post-hoc t-tests on the regional values. *p < 0.05, **p < 0.01, ***p < 0.001.

Compared to HC, MSA patients showed reduced WM volume in a cluster extending from the cerebellar WM to the brainstem (cluster size = 413,700 voxels, Fig. 2a). When compared to PD patients, MSA showed a WM volume reduction in a cluster extending from the cerebellar WM to the brainstem (cluster size = 257,580 voxels, Fig. 2b). No significant differences were detected when comparing PD versus HC. Regional post-hoc analyses showed a significant difference in WM atrophy of the cerebellum/brainstem cluster between MSA-P and both PD and HC and between MSA-C and both PD and HC (Fig. 2c). Regional analysis on SN revealed significant differences in left SN between HC and MSA (HC vs MSA-C, *p* < 0.001; HC vs MSA-P, *p* = 0.017), and between PD and MSA (PD vs MSA-C, *p* < 0.001; PD vs MSA-P, *p* = 0.018). Significant differences were also detected in right SN between HC and MSA (HC vs MSA-C, *p* < 0.001; HC vs MSA-P, *p* = 0.008), and between PD and MSA (PD vs MSA-C, *p* < 0.001; PD vs MSA-P, *p* = 0.005), see Supplementary Fig. 1.

Four HC, 1 PD and 3 MSA patients were excluded from the T1w/T2w imaging analysis due to missing 3D T2w series.

**Figure 3:**
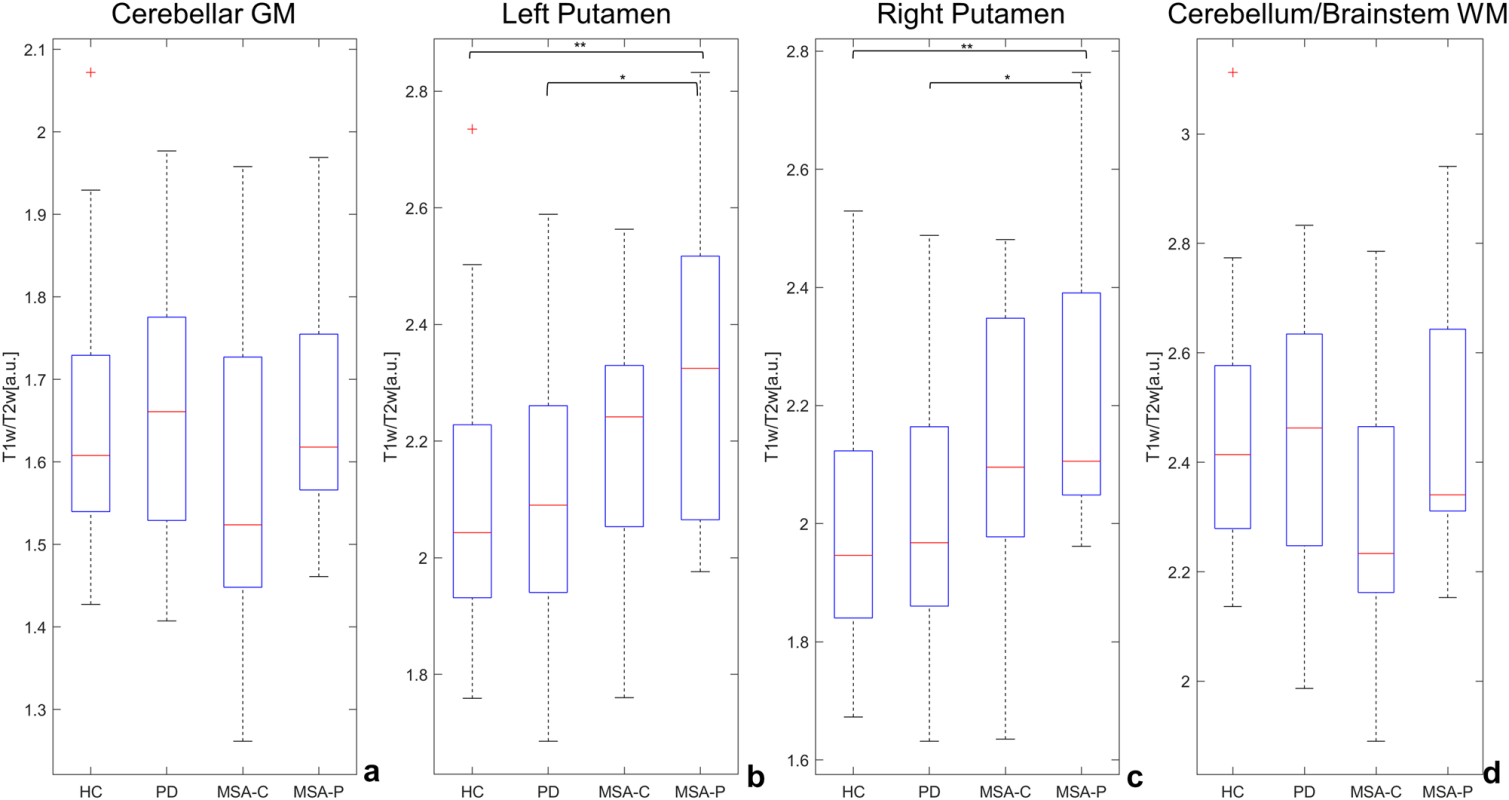
Box-plots of the T1w/T2w distribution in the significant clusters (from the comparison HC vs MSA in GM and WM) for each group, and results of the post-hoc t-tests on the regional values. *p < 0.05, **p < 0.01.

Whole brain voxel-based comparison of T1w/T2w maps did not show any difference between groups. Nonetheless, regional post-hoc analysis in the aforementioned clusters of GM or WM atrophy showed significant differences between MSA-P and HC and between MSA-P and PD in the right and left putamen, with increased T1w/T2w values in the MSA-P subgroup (see Fig. 3b, c). No significant differences in terms of T1w/T2w values were detected in the clusters of GM atrophy in the cerebellum and WM atrophy in the cerebellum/brainstem (Fig. 3a, d). The analysis on regional T1w/T2w mean values in SN disclosed a significant difference between HC and MSA-C subgroup (*p* = 0.01) for left SN and a significant difference between HC and PD (*p* = 0.03) and HC and MSA-C subgroup (*p* < 0.001) in right SN, see Supplementary Fig. 1.

Regional values in significantly different clusters were also used for discriminant analysis between groups by using both T1w/T2w parameters and combined GM or WM with T1w/T2w.

T1w/T2w value in the left putamen discriminated MSA versus HC, and MSA versus PD, but not MSA-P versus PD. T1w/T2w value in the right putamen discriminated MSA versus HC and MSA versus PD, but not MSA-P versus PD. T1w/T2w value in left and right SN discriminated significantly between MSA and HC groups.

Combination of regional GM and T1w/T2w in the cerebellum significantly discriminated MSA from HC and MSA from PD, but not MSA-P from PD. The multi-parameter (multi-variate) combination of regional GM and T1w/T2w in the left putamen significantly discriminated MSA from HC, MSA from PD and MSA-P from PD. The multi-parameter (multi-variate) combination of regional GM and T1w/T2w in the right putamen significantly discriminated MSA from HC, MSA from PD, and MSA-P from PD. The multi-parameter (multi-variate) combination of regional WM and T1w/T2w in the cerebellum/brainstem significantly discriminated MSA from HC, MSA from PD, but not MSA-P from PD. Additionally the combination of WM and T1w/T2w regionally in left and right SN allows to significantly discriminate between MSA and HC, MSA and PD and MSA-C subgroup and PD. The detailed results of the ROC analysis (AUC, sensitivity, specificity and *p*-values) are summarized in Table 2.

**Table 2:** ROC analysis results.

	T1w/T2w				T1w/T2w + Tissue density (GM or WM)			
	AUC	*p*	Sensitivity	Specificity	AUC	*p*	Sensitivity	Specificity
**MSA vs HC**								
L-Putamen	**0.70**	**0.005**	**0.87**	**0.46**	**0.84**	** < 0.001**	**0.74**	**0.83**
R-Putamen	**0.71**	**0.003**	**0.91**	**0.50**	**0.88**	** < 0.001**	**0.78**	**0.87**
Cerebellum GM	0.54	> 0.05	0.30	0.88	**0.93**	** < 0.001**	**0.78**	**0.96**
Brainstem WM	0.60	> 0.05	0.65	0.62	**0.88**	** < 0.001**	**0.83**	**0.75**
L-SN	**0.72**	** < 0.001**	**0.61**	**0.77**	**0.88**	** < 0.001**	**0.96**	**0.71**
R-SN	**0.76**	** < 0.001**	**0.87**	**0.60**	**0.90**	** < 0.001**	**0.96**	**0.77**
**MSA vs PD**								
L-Putamen	**0.67**	**0.019**	**0.52**	**0.81**	**0.78**	** < 0.001**	**0.57**	**0.92**
R-Putamen	**0.68**	**0.010**	**0.83**	**0.56**	**0.83**	** < 0.001**	**0.78**	**0.83**
Cerebellum GM	0.57	> 0.05	0.48	0.56	**0.85**	** < 0.001**	**0.74**	**0.98**
Brainstem WM	0.59	> 0.05	0.52	0.65	**0.81**	** < 0.001**	**0.61**	**0.90**
L-SN	0.63	> 0.05	0.43	0.87	**0.79**	** < 0.001**	**0.96**	**0.62**
R-SN	0.64	> 0.05	0.26	0.94	**0.80**	** < 0.001**	**0.65**	**0.90**
**MSA-P vs PD**								
L-Putamen	0.72	> 0.05	0.30	0.92	**0.89**	**0.01**	**0.22**	**1**
R-Putamen	0.66	> 0.05	0.57	0.63	**0.93**	** < 0.001**	**0.26**	**1**
Cerebellum GM	0.40	> 0.05	0.52	0.52	0.68	> 0.05	0.78	0.88
Brainstem WM	0.35	> 0.05	0.70	0.44	0.61	> 0.05	0.39	1
L-SN	0.62	> 0.05	0.57	0.81	0.78	> 0.05	0.48	0.94
R-SN	0.45	> 0.05	0.87	0.58	0.76	> 0.05	0.61	0.92
**MSA-C vs PD**								
L-Putamen	0.50	> 0.05	0.52	0.75	0.59	> 0.05	0.83	0.58
R-Putamen	0.48	> 0.05	0.17	0.98	0.65	> 0.05	0.78	0.87
Cerebellum GM	0.68	> 0.05	0.13	0.92	**0.92**	** < 0.001**	**0.57**	**1**
Brainstem WM	**0.76**	**0.018**	**0.26**	**0.88**	**0.93**	** < 0.001**	**0.48**	**1**
L-SN	0.62	> 0.05	0.26	0.92	**0.84**	** < 0.001**	**0.52**	**0.87**
R-SN	0.66	> 0.05	0.22	0.96	**0.84**	** < 0.001**	**0.52**	**0.94**

ROC analysis results for patients’ discrimination. T1w/T2w regional values were used to discriminate between groups both alone and in combination with mean regional tissue density (GM/WM). Significant results were enhanced in bold.

After correcting regional GM or WM tissue density for age and sex, residual GM volume in left and right Putamen was significantly correlated with the UMSARS falling score in all the MSA group (left: ρ =  − 0.379, *p* = 0.05; right ρ =  − 0.511, *p* = 0.008). Only in MSA-P patients residual GM volume in the cerebellum/brainstem was significantly correlated with UMSARS-II (ρ =  − 0.515, *p* = 0.05) and UMSARS falling score (ρ =  − 0.529, *p* = 0.05) while residual WM volume in the cerebellum/brainstem was significantly correlated with UMSARS-I (ρ =  − 0.560, *p* = 0.037), UMSARS-II (ρ =  − 0.537, *p* = 0.047), UMSARS walking (ρ =  − 0.544, *p* = 0.044) and UMSARS falling (ρ =  − 0.645, *p* = 0.013) scores. Regional WM density in bilateral SN was significantly correlated to UMSARS walking score (left: ρ =  − 0.631, *p* = 0.015; right ρ =  − 0.593, *p* = 0.025) and UMSARS falling score (left: ρ =  − 0.573, *p* = 0.032; right ρ =  − 0.550, *p* = 0.041) only in the MSA-P subgroup. After correcting T1w/T2w regional values for age and sex, significant correlations were found between UMSARS autonomic score and regional value in left SN (ρ =  − 0.776, *p* = 0.0049) only in the MSA-P subgroup. None of the correlations resulted significant after correction for multiple comparisons.

## Discussion

In this study we performed a multi-parametric evaluation of tissue integrity assessing both T1w/T2w ratio images and voxel-based morphometry on GM and WM tissue density to investigate whole brain damage in MSA patients as compared to PD patients and HC and evaluate a potential semi-quantitative biomarker for differential diagnosis with PD.

As expected, we found MSA patients to have GM atrophy in bilateral putamen and cerebellum and WM atrophy in the cerebellum/brainstem compared to both PD patients and HC. Post-hoc regional analysis showed that MSA patients had significant bilateral atrophy of SN pars compacta as compared to both PD and HC. This finding is consistent with neuropathological findings of MSA^3^ and previous VBM data on gray matter atrophy in dorsal midbrain^17^, but in our study we first obtained a semiquantitative assessment of regional tissue density in the SN of MSA patients considering WM density and T1w/T2w values.

On the other hand, T1w/T2w changes did not strictly parallel the obtained atrophy patterns, with MSA and PD patients showing similar values in the cerebellum/brainstem, but MSA-P showing a significant increase in T1w/T2w values in both right and left putamen as compared to PD and HC. Moreover, T1w/T2w regional mean value in bilateral SN pars compacta was found to be higher in MSA-C patients compared to HC, suggesting that T1w/T2w ratios deserve to be further studied as an MRI biomarker in both MSA subtypes. Additionally, T1w/T2w regional mean value in the right SN was found to be higher in PD patients compared to HC, confirming recent evidence in PD patients^12^. The combined use of regional GM and T1w/T2w values in the right and left putamen showed the highest accuracy in discriminating MSA-P from PD, while the combined use of the same parameters showed a good accuracy in discriminating MSA from both PD and HC. A similar good accuracy was also found in discriminating MSA from PD and HC by either combining regional GM and T1w/T2w values in the cerebellum or regional WM and T1w/T2w in the cerebellum and brainstem. Finally, a quite good accuracy was found in discriminating MSA from PD and HC, and MSA-C from PD by combining regional tissue density and T1w/T2w values in both left and right SN.

The higher degree of putaminal and cerebellar gray matter atrophy of MSA patients largely confirm previous results^17–19^ and is in keeping with the evidence of MSA being a more aggressive and widespread disorder than PD. We also found a significant WM volume reduction in a cluster extending from the cerebellum to the brainstem in both MSA-P and MSA-C versus PD and HC. Consistently, few previous studies have already described WM atrophy in brainstem and cerebellum in MSA-C and MSA-P patients as compared to HC^20–22^. In patients with PD we found no differences in terms of atrophy when compared to HC, which is also in line with previous results obtained in patients with similar disease duration^23^.

Previous studies have used the T1w/T2w ratio to detect cortical changes in patients with multiple sclerosis, showing lower values in pathologically vulnerable regions^10,11^.

On the other hand, higher cortical values of the T1w/T2w ratio have been found in Alzheimer’s disease^24^ and Huntington’s disease^25^ as compared to HC, and a similar finding has been reported in a subcortical structure, namely substantia nigra pars compacta, in PD patients compared to HC^12^. Decrease in neuronal density, resulting in higher myelin proportions, or iron accumulation have been suggested to account for such previous results^25^ and may also account for the increased T1w/T2w ratio found in the atrophic putamen of our MSA-P patients. Indeed, putaminal diffusivity changes have been reported corresponding to prominent neuronal loss^26^, and quantitative susceptibility mapping has confirmed increased iron deposition in the putamen of MSA-P patients^27,28^.

More recently, a reduced T1w/T2w ratio in the medium cerebellar peduncle has been reported in MSA-C compared to HC^29^, suggesting that this measure can also be useful as a quantitative biomarker of myelin loss. Indeed, this measure was also found to be correlated with the ICARS score in early MSA-C but, consistently with our results, not with UMSARS part 2 scores, possibly due to the inadequacy of the latter scale that was originally developed to characterize cerebellar ataxia and parkinsonism^29^. There are, however, some important conceptual and methodological differences between this study and ours: in fact, differently from the previous study, we adhered to the original protocol described to assess T1w/T2w ratio^6^, using 3D acquisition schemes and 1 mm^3^ spatial resolution in both T1w and T2w images acquisition. This protocol was specifically developed to analyze the entire brain, i.e., without prior assumptions on the potentially affected regions. To this more general purpose, we combined the T1w/T2w analysis with a classical VBM analysis at the same (typical) resolution of morphometric studies at 3 Tesla.

We found that UMSARS motor scores in MSA-P patients were worse in patients with greater GM and WM atrophy in the cerebellum/brainstem and, similarly, UMSARS functional scores were worse in patients with greater WM atrophy in the cerebellum/brainstem. We can speculate that correlations with such clinical parameters may reflect the progressive brainstem/cerebellar involvement during the course of MSA-P. Consistently, a similar relationship between UMSARS motor scores and cerebellar atrophy in MSA-P patients has been reported in a previous VBM study, also failing to show relationships between UMSARS scores and putaminal atrophy^30^. By looking at specific items from the UMSARS I, higher falling scores were related with bilateral putaminal atrophy in the whole MSA group, greater GM and WM atrophy in the cerebellum/brainstem only in MSA-P patients and greater WM atrophy in the SN. Moreover, greater impairment in walking was associated with greater WM atrophy in the cerebellum/brainstem and bilateral SN only in MSA-P patients. Indeed, none of such correlations with specific UMSARS items survived after correction for multiple comparisons, due to the great number of variables examined, nonetheless these functional correlates may be of interest for future studies. We must acknowledge that evaluating our MSA patients also with UPDRS and an ataxia specific scale would have been desirable to better evaluate relationships with MRI parameters.

Further validation of the T1w/T2w ratio in MSA is needed to establish if this measure alone or combined with other validated MRI parameters can be useful as a quantitative MRI biomarker for differential diagnosis and follow-up of MSA patients.

## Supplementary Information

Supplementary Figure 1.
