# Supplementary material for: Magnetic resonance T1w/T2w ratio and voxel-based morphometry in multiple system atrophy
Source: Sci Rep. 2021 Nov 4;11:21683. doi: 10.1038/s41598-021-01222-5 (PMC8569168; doi:10.1038/s41598-021-01222-5)
Supplement: Supplementary file 1 — Supplementary Figure 1. [file 41598_2021_1222_MOESM1_ESM.pdf]

Magnetic resonance T1w/T2w ratio and voxel-based morphometry in multiple system atrophy

Ponticorvo S, [1] **Manara R,** [2] **Russillo MC,** [1] **Erro R,** [1] **Picillo M,** [1] **Di Salle G,** [3] **Di Salle F,** [1] **Barone P,** [1] **Esposito F,** [4] **Pellecchia MT**[1]

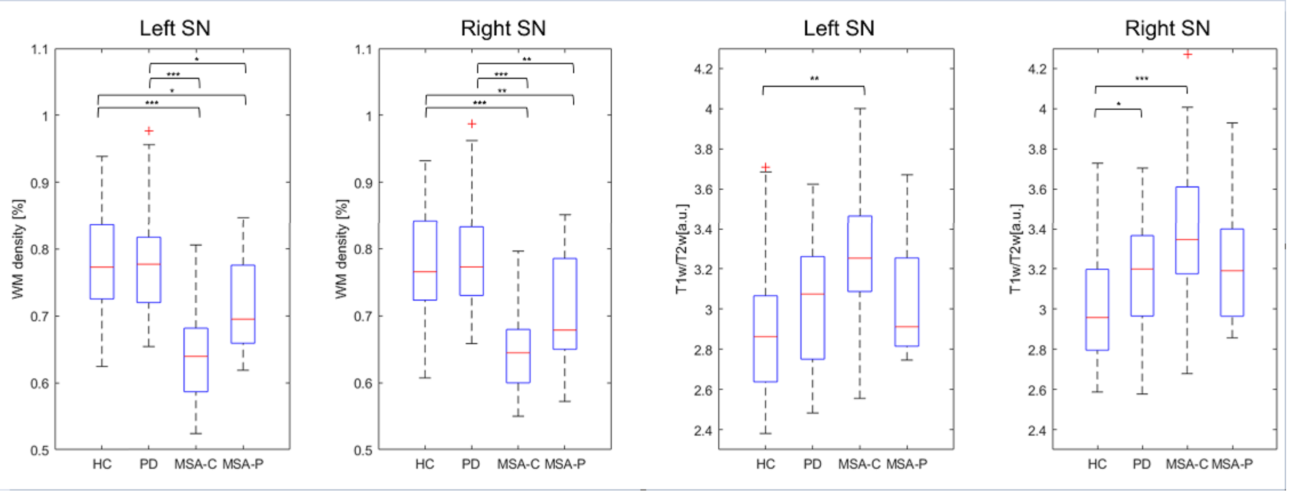

Supplementary Figure 1. Box-plots of the WM density (first and second boxes) and T1w/T2w (third and fourth boxes) regional mean values distribution in the bilateral substantia nigra pars compacta (SN) for each group. Significant differences are indicated with asterisks (*p<0.05, **p<0.01).
